# Stochastic Syncing in Sinusoidally Driven Atomic Orbital Memory

**DOI:** 10.1021/acsnano.3c09635

**Published:** 2024-01-30

**Authors:** Werner M. J. van Weerdenburg, Hermann Osterhage, Ruben Christianen, Kira Junghans, Eduardo Domínguez, Hilbert J. Kappen, Alexander Ako Khajetoorians

**Affiliations:** † Institute for Molecules and Materials, Radboud University, 6525 AJ Nijmegen, The Netherlands; ‡ Donders Institute for Neuroscience, Radboud University, 6525 AJ Nijmegen, The Netherlands

**Keywords:** orbital memory, scanning tunneling microscopy, dopants on semiconductors, stochastic dynamics, synchronization, black phosphorus, neuromorphic computing

## Abstract

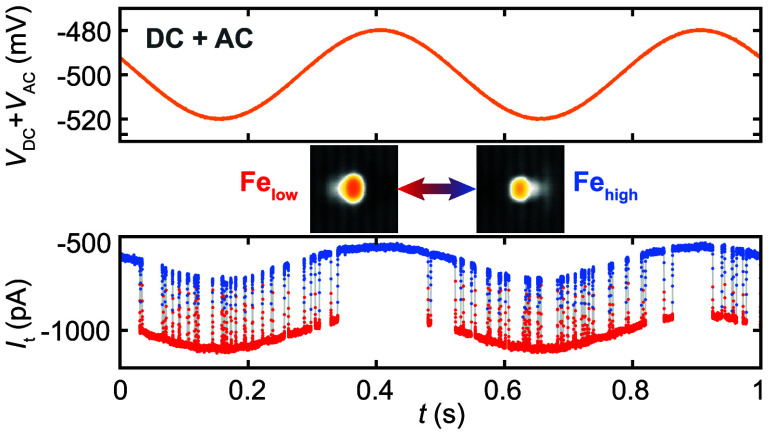

Stochastically fluctuating multiwell systems are a promising route toward physical implementations of energy-based machine learning and neuromorphic hardware. One of the challenges is finding tunable material platforms that exhibit such multiwell behavior and understanding how complex dynamic input signals influence their stochastic response. One such platform is the recently discovered atomic Boltzmann machine, where each stochastic unit is represented by a binary orbital memory state of an individual atom. Here, we investigate the stochastic response of binary orbital memory states to sinusoidal input voltages. Using scanning tunneling microscopy, we investigated orbital memory derived from individual Fe and Co atoms on black phosphorus. We quantify the state residence times as a function of various input parameters such as frequency, amplitude, and offset voltage. The state residence times for both species, when driven by a sinusoidal signal, exhibit synchronization that can be quantitatively modeled by a Poisson process based on the switching rates in the absence of a sinusoidal signal. For individual Fe atoms, we also observe a frequency-dependent response of the state favorability, which can be tuned by the input parameters. In contrast to Fe, there is no significant frequency dependence in the state favorability for individual Co atoms. Based on the Poisson model, the difference in the response of the state favorability can be traced to the difference in the voltage-dependent switching rates of the two different species. This platform provides a tunable way to induce population changes in stochastic systems and provides a foundation toward understanding driven stochastic multiwell systems.

The development of neuromorphic hardware that is inherently adaptive and exhibits self-learning requires a deeper understanding of materials that show multiwell behavior.^1^ One route toward this end is to create material systems with tunable multiwell energy landscapes, where local minima represent information.^2^ Such systems are mimicked by energy-based models in machine learning: for instance the Hopfield model or its stochastic analogue, the Boltzmann machine, where the energy landscape can be described by an Ising Hamiltonian.^3−5^ In this way, it was proposed^6^ that coupled atomic spins on surfaces^7−11^ can be used to realize the prerequisite multiwell energy landscapes, in the presence of long-range competing interactions. Recently based on this concept, it was shown that arrays of coupled binary orbital memory states^12−14^ emulate an atomic-scale Boltzmann machine, by exploiting their dynamical properties.^13,15^ The multistate dynamics emerging from the coupling between stochastic atoms directly maps onto the Hamiltonian of the Boltzmann machine, with tunable stochastic weights. However, it is still unclear how such stochastic multiwell systems respond to a dynamical drive signal, an important prerequisite toward computational functionality.

A necessary step toward understanding the dynamic response of stochastic multiwell systems is the development of experimental and theoretical model systems that quantify the dynamical response of binary spins in response to the rich parameter space available.^16^ For an individual binary spin in the stochastic regime, the response to a periodic stimulus is often described in terms of phase-synchronization and stochastic resonance (SR).^17−19^ Generally, SR manifests itself in nonlinear dynamical systems as an enhanced response to a weak input signal due to the presence of intermediate noise compared to the response for low and high noise amplitudes. In systems with an activation energy, SR arises when a weak, noisy signal periodically exceeds the threshold, thereby inducing dynamics. This is referred to as threshold SR.^18^ However, in Arrhenius-type double-well systems where the switching rates are always finite, similar phenomena can be observed due to a periodic modulation of the switching rates, known as dynamical SR.^17,18^ Examples of two-state systems where this effect was observed are charging and discharging of a quantum dot^20^ and adsorption states of a hydrogen molecule.^21^ Recently, dynamical SR has also been reported in an atomic spin system.^22^ Complementary to such atomic systems based solely on spin,^10,23−25^ atomic orbital memory provides a richer parameter space where all the relevant transition rates can be tuned by input parameters as well as the possibility to realize multiwell potential landscapes. Since the dynamical complexity rapidly increases with the number of stochastic units,^15^ it is essential to first gain a fundamental understanding of how synchronization and frequency-induced population coding can be achieved with AC signals for an individual bistable orbital memory atom.

Here, we quantify the stochastic response of orbital memory derived from individual Fe and Co atoms on black phosphorus (BP) to sinusoidal input voltages. Using scanning tunneling microscopy (STM), we applied a chosen DC bias voltage and AC sinusoidal voltage and studied the stochastic response of the orbital states of individual Fe and Co atoms as a function of frequency and amplitude. For individual Fe atoms, we observe a frequency-dependent change in the state-dependent residence times. This modulation results from the synchronization between the input signal and the switching events. Concomitantly, we find a change in the extracted state favorability, namely, the asymmetry, near the frequency corresponding to the mean residence time of the two orbital states of Fe. In contrast, we do not observe a frequency-dependent change in the asymmetry for individual Co atoms, even though there is a significant synchronization response in the measured residence time statistics. The origin of this effect is related to the voltage-dependent switching rates of each system. To quantify this effect, we measure the change in asymmetry as a function of the DC bias voltage, or namely, the voltage susceptibility, for both Fe and Co. We relate the synchronization behavior and frequency-dependent changes in the asymmetry to the voltage susceptibility in the DC limit. Based on a stochastic model, we demonstrate that these observations are dictated by the switching rates for each state in the DC limit for the given atom. This result illustrates that detection schemes based on synchronization-induced population changes are insensitive to binary spins with low voltage susceptibility. The orbital memory platform additionally enables fine-tuning of the frequency-induced stochastic behavior.

## Results and Discussion

**Figure 1:**
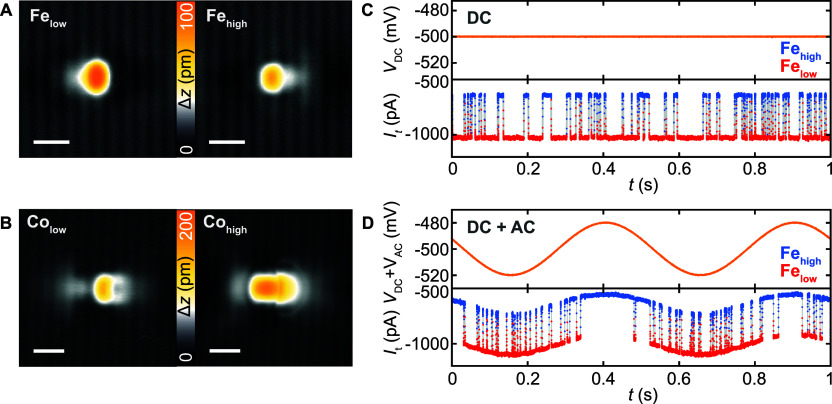
Stochastically switching orbital memory states of Fe and Co atoms on black phosphorus. (A, B) Constant-current STM images of (A) an individual Fe atom and (B) an individual Co atom adsorbed in the hollow site of BP, imaged for both orbital memory states (low/high) (*V*_DC_ = −400 mV; (A) *I*_t_ = 10 pA and (B) 50 pA; scale bar 1 nm). (C, D) Telegraph noise recorded at the center of an Fe atom in constant height for (C) *V*_AC_ = 0 mV, *V*_DC_ = −500 mV and (D) *V*_AC_ = 40 mV, *V*_DC_ = −500 mV, *f* = 2 Hz. The tip was stabilized on the substrate at *V*_DC_ = −400 mV and *I*_t_ = 100 pA, before the feedback loop was opened.

We performed scanning tunneling microscopy and spectroscopy (STM/STS) on Fe and Co atoms adsorbed on a black phosphorus (BP) surface using a home-built UHV-STM system^26^ (*p* < 5 × 10^–10^ mbar), operating at a temperature of *T* ≈ 7 K. Individual Fe and Co atoms, residing in the hollow site of BP, exhibit orbital memory: a bistability in their valency.^12,13^ The two stable valency configurations can be distinguished in constant-current STM images by both their apparent height (Δ*z*), and the shape of their charge density (Figure 1A,B). We label each of these two distinguishable states as high/low for each atomic species. Below a threshold DC voltage, each orbital state remains indefinitely stable, allowing for state identification.

Above an applied DC voltage (*V*_DC_), there is a finite probability of switching between the two orbital configurations for both individual Co and Fe atoms.^12,13^ The stochastic switching is recorded by measuring the tunneling current (*I*_t_) as a function of time for a constant tip–sample separation and lateral tip position. Before each measurement, the tip is first stabilized on the BP substrate to a predefined tip–sample separation. Figure 1C exemplifies stochastic switching for an individual Fe atom (Fe_low_/Fe_high_). By recording a statistically significant number of stochastic switching events (>1000 events), we extract the average residence time () of each orbital state, where τ_*i*_ refers to one measured residence time with index *i* and *N*_low/high_ is the total number of detected switching events for a given state (see Figure S3 for details about the data analysis). Moreover, from the average residence times, we extract the asymmetry *A* = (τ̅_low_ – τ̅_high_)/(τ̅_low_ + τ̅_high_), which measures the state favorability. The values of τ̅_low/high_ and *A* are sensitive to *V*_DC_, *I*_t_, and the position of the tip. We selectively chose parameters to yield switching rates within the time resolution of the experiment (see Figure S2) and avoided the strongly asymmetric regime for these experiments.

In addition to measuring the stochastic switching for a given *V*_DC_, we also measured the stochastic switching in response to an additional sinusoidal voltage *V*_AC_ = |*V*_AC_|/2 sin(2π*ft*) added to *V*_DC_ (Figure S2). |*V*_AC_| is subsequently defined as the peak-to-peak amplitude. The resultant values of *I*_t_ are modulated, as shown in Figure 1D, for the case where |*V*_AC_| = 40 mV and *f* = 2 Hz. The number of switching events is highest when |*V*_DC_ + *V*_AC_| is maximized, indicating that the switching probability of each state *i* is sensitive to the phase φ of *V*_AC_. Moreover, the switching probability remains finite at any point in the waveform. This means that we measure synchronization of a thermodynamic double-well system to a sinusoidal input signal.^17,18^

**Figure 2:**
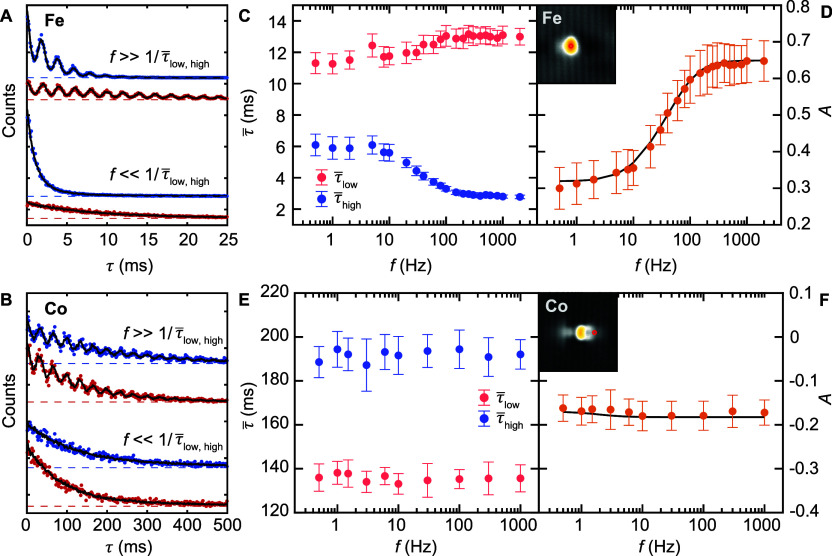
Frequency dependence of mean residence times. (A, B) Histogram plots of the detected τ_*i*_ for (A) Fe_low_ (red) and Fe_high_ (blue) and (B) Co_low_ (red) and Co_high_ (blue) for two frequencies, namely (A) *f* = 1 and 500 Hz and (B) *f* = 1 and 30 Hz, measured with (A) *V*_DC_ = −500 mV and (B) *V*_DC_ = 480 mV with an amplitude of |*V*_AC_| = 40 mV (peak-to-peak). The histograms present ≥28 extracted residence times per bin, i.e. per data point (plotted with an artificial vertical offset (dashed lines) for each state and frequency). Histograms of synthesized stochastic data based on a Poisson point process are shown in black. (C, D) Time-averaged residence time τ̅_low/high_ and deduced state asymmetry *A* of Fe_low_ (red) and Fe_high_ (blue) as a function of the frequency of *V*_AC_. (E, F) Time-averaged residence time τ̅_low/high_ and deduced state asymmetry *A* of Co_low_ (red) and Co_high_ (blue) as a function of the frequency of *V*_AC_. Solid lines in (D) and (F) represent the simulated frequency response, based on rate equations (see Supplementary text S1). The tip was stabilized on the substrate at *V*_DC_ = −400 mV and (A, C, D) *I*_t_ = 100 pA or (B, E, F) *I*_t_ = 30 pA.

In Figure 2A,B, we illustrate histograms of the state-resolved τ_*i*_ for a set of frequencies, for both atom types (|*V*_AC_| = 40 mV). Each data point represents the number of detected τ_*i*_ values within a given time interval. The histograms contain the exponential decay behavior expected for a Poisson process. In addition to the exponential decay, above a given frequency, there are clear deviations from the pure exponential distribution, and additional modulations emerge on top of the exponential decay. Such features are typical for phase synchronization in a stochastic system, indicating that switching preferentially occurs around one well-defined phase φ. This results in a higher favorability of residence times with τ_*i*_*∼ n*/*f*, where *n* is an integer. Such features are also present in systems that exhibit SR^17^ and have been observed for atomic spins derived from Fe atoms.^22^ For individual Co atoms, we also observe synchronization for both states, albeit with fewer statistics, given the longer residence times of the Co system. For a given |*V*_AC_|, the amplitude of the modulations depends on the specific state and atom type.

In order to further quantify the stochastic response, we extracted the values of τ̅_low_, τ̅_high_, and *A* as a function of *f* (Figure 2C,D). For Fe, *A*(*f*) illustrates a strong change near the frequency *f*_Fe_^*^ ≈ 1/τ̅_av_, where τ̅_av_ corresponds to the average residence time of both states at the applied value of *V*_DC_. Above *f*_Fe_^*^, τ̅_low_ and τ̅_high_ increase and decrease, respectively, and saturate to the asymptotic values. Since the change in *A*(*f*) leads to a change in the time-averaged occupation and each orbital state is distinguished by a different tunneling current, the frequency response of individual Fe atoms can be detected in the time-averaged current signal (see Figure S9). Therefore, the change in *A*(*f*) represents the time-averaged frequency response of the system. In contrast to the significant frequency response of both τ̅ and *A* for an Fe atom, there is no significant frequency response in the various extracted variables for a Co atom, as plotted in Figure 2E,F. We explain this difference below.

**Figure 3:**
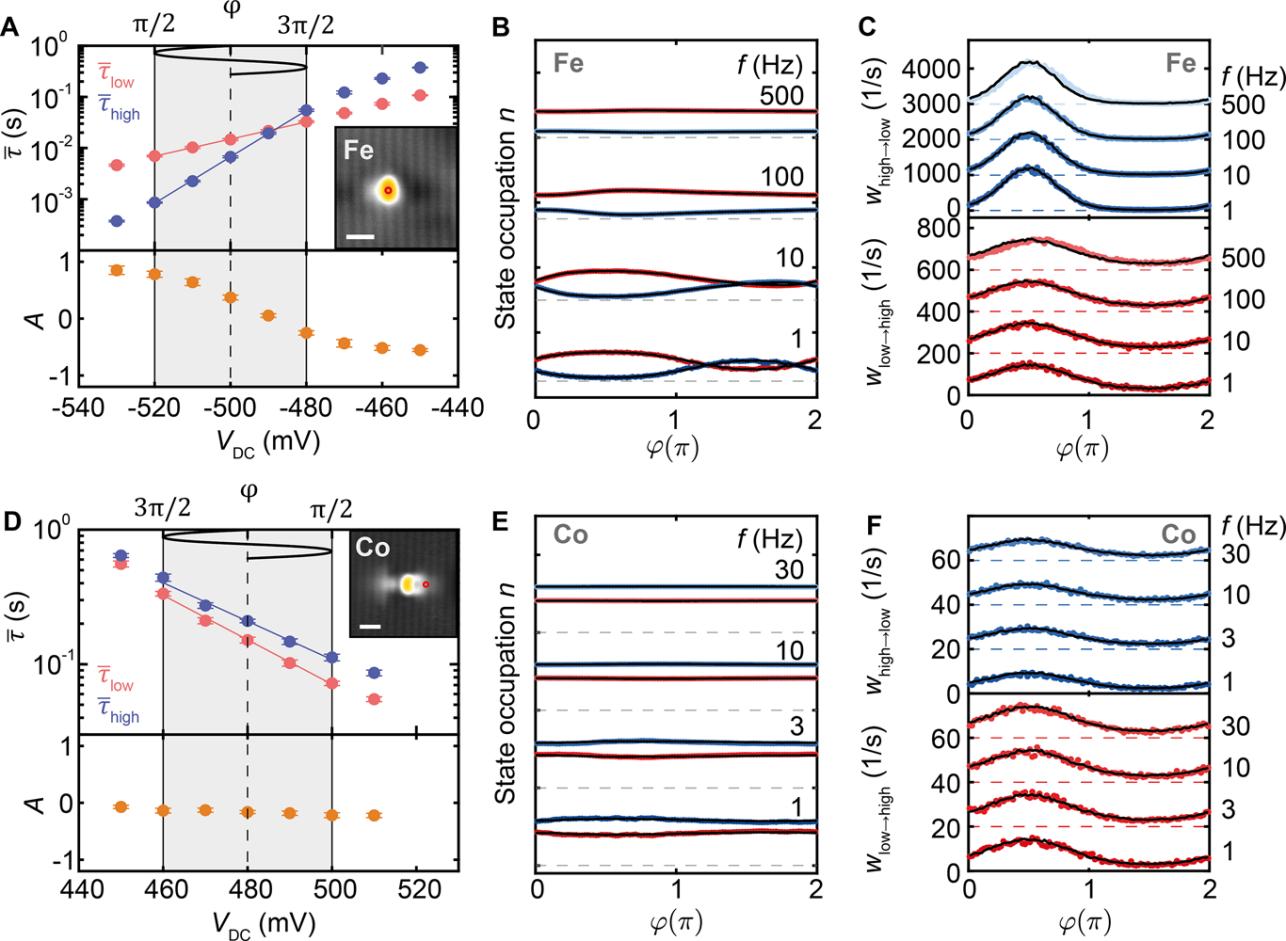
Voltage dependence of state asymmetry. (A) Average residence times τ̅_low/high_ for Fe_low_ (red) and Fe_high_ (blue) and extracted asymmetry *A* (lower panel) as a function of bias voltage *V*_DC_, measured with *V*_AC_ = 0 mV on top of an individual Fe atom (see inset; scale bar 1 nm). Solid lines represent exponential fits. (B) Phase-resolved state occupation *n*_low_ and *n*_high_ (artificial offset, dashed gray lines; simulation, solid black lines) and (C) conditional switching rates *w*_low→high_ and *w*_high→low_ for different frequencies (artificially offset, dashed lines; simulation, solid black line), averaged over many periods. |*V*_AC_| is indicated by the gray shaded area, and the phase φ is defined at the top of (A). The tip height was stabilized on the substrate at *V*_DC_ = −400 mV and *I*_t_ = 100 pA. (D) Average residence times τ̅_low/high_ for Co_low_ (red) and Co_high_ (blue) and extracted asymmetry *A* (lower panel) as a function of bias voltage *V*_DC_, measured with *V*_AC_ = 0 mV next to an individual Co atom (see inset; scale bar 1 nm). Solid lines represent exponential fits. (E) Phase-resolved state occupation *n*_low_ and *n*_high_ (artificial offset, dashed gray lines; simulation, solid black lines) and (F) conditional switching rates *w*_low→high_ and *w*_high→low_ for different frequencies (artificially offset, dashed lines; simulation, solid black lines), averaged over many periods. |*V*_AC_| is indicated by the gray shaded area, and the phase φ is defined at the top of (D). The tip height was stabilized on the substrate at *V*_DC_ = −400 mV and *I*_t_ = 30 pA.

In order to understand the difference in the frequency response between Fe and Co atoms, we first quantified τ̅_low/high_ as a function of *V*_DC_ without an additional *V*_AC_ signal (Figure 3A,D). The general *V*_DC_-dependent trend for both Co and Fe agrees with previous publications,^12,13^ where τ̅_low/high_ decreases for an increasing magnitude of |*V*_DC_|. Phenomenologically, we can describe τ̅_low/high_(*V*_DC_) in the voltage range of interest using an exponential dependence, as shown with the fits in Figure 3A,D. In addition, we also extracted the dependence of *A* on *V*_DC_ and find that *A*(*V*_DC_) in the studied range strongly depends on the value of *V*_DC_ for the case of Fe, but it is nearly independent of *V*_DC_ for Co. Accordingly, we can relate the slope of *A*, which we define as χ = ∂A/∂*V*_DC_. We refer to χ as the voltage susceptibility. From Figure 3A,D, we observe that |χ_Fe_| ≫ |χ_Co_|, which, as we show below, is directly linked to the frequency response. Besides τ̅ and *A*, the switching characteristics can be described in terms of the state occupations, defined as *n*_low_ = τ̅_low_/(τ̅_low_ + τ̅_high_) and *n*_high_ = τ̅_high_/(τ̅_low_ + τ̅_high_).

We subsequently model the stochastic response of each atom type to *V*_AC_. We assume that τ_*i*_ in the absence of *V*_AC_ is described by a Poisson process with an exponential probability distribution (see Figure S3B). For *V*_AC_ = 0, the distribution of τ_*i*_ can be described by average transition rates *w*_low→high_(*V*_DC_) = 1/(τ̅_low_(*V*_DC_)) and *w*_high→low_(*V*_DC_) = 1/(τ̅_high_(*V*_DC_)), which are extracted from Figure 3A,D. Subsequently, we simulate the stochastic dynamics in response to a nonzero value of *V*_AC_, considering a Poisson point process with periodically changing *w* (eqs S2 and S3), as introduced in ref (27). Since *A*(*f*) is a time-averaged quantity, it can be simulated by solving the associated rate equations (see eqs S4–S6). We find that the simulated *A*(*f*) (solid line) is in good agreement with the experimental data in Figure 2D,F. To model the τ_*i*_ histograms, stochastic data can be generated using the experimentally determined *w*_low→high_(*V*_DC_) and *w*_high→low_(*V*_DC_) with the aforementioned assumptions (see Supplementary text S2). This approach allows us to model the τ_*i*_ histograms (Figure 2A,B), as well as *n*_low_, *n*_high_, *w*_low→high_, and *w*_high→low_ as a function of the modulation phase φ (Figure 3).

Based on this modeling, we considered the average *n*_low_, *n*_high_, *w*_low→high_, and *w*_high→low_ within a period of the waveform. In Figure 3B, we plot the phase-resolved *n*_low_(φ) (red) and *n*_high_(φ) (blue) for various frequencies. Each data point is extracted by subdividing one period of the waveform into 100 phase segments, calculating *n*_low_ and *n*_high_ for each segment, and averaging over many periods. For *f* ≪ f*, *n*_low_ and *n*_high_ are clearly modulated, directly following the changes of *A*(*V*_DC_) in Figure 3A. For instance, at φ = 3/2 π and *f* = 1 Hz, the Fe_high_ state is more favorable, partially compensating for the larger *n*_low_ in the first half of the waveform. With increasing *f*, the modulation of *n*_low_ and *n*_high_ becomes less pronounced and the maximum of *n*_high_ shifts to larger φ, indicating that *n*_low_ and *n*_high_ exhibit a delayed response in time to *V*_AC_. For *f* ≫ *f*_Fe_^*^, *n*_low_ and *n*_high_ show a flat response as a function of φ, converging to a time-averaged *A*. We find that the evolution of *n*_low_ and *n*_high_ as a function of *f* is reproduced in the stochastic model calculations. The phase-resolved conditional transition rates, *w*_low→high_(φ) and *w*_high→low_(φ), can be determined from the switching rates within each phase segment, divided by the occupation of the corresponding state in that phase segment.^22^ As shown in Figure 3C, most of the switches occur around φ = π/2, as expected from τ̅_low/high_ in the voltage range *V*_DC_ ± |*V*_AC_|/2, and *w*_low→high_(φ) and *w*_high→low_(φ) are approximately constant as a function of *f*. The phase-resolved analysis for individual Co atoms reveals that *w*_low→high_(φ) and *w*_high→low_(φ) (Figure 3F) are clearly modulated, while *n*_low_ and *n*_high_ (Figure 3E) show negligible changes within one period of the waveform. This can be understood as a direct consequence of the parallel evolution of τ̅_low_ and τ̅_high_; namely χ_Co_ ≈ 0 for the two Co states in Figure 3D. As a result, the frequency-induced change of *A*(*f*) (Figure 2E,F) is zero for Co atoms.

Based on the comparison between the dynamical properties of Fe and Co atoms and supported by stochastic modeling, we can identify the requirements to observe a time-averaged frequency response and link it to the measured τ̅_low/high_(*V*_DC_). Namely, the frequency-induced changes in *A*(*f*) can only be observed if *n*_low_ and *n*_high_ are modulated for *f* ≪ f*. This can only be obtained when *w*_low→high_(*V*) and *w*_high→low_(*V*) evolve such that *A*(*V*) exhibits significant changes in the voltage range of *V*_DC_ ± |*V*_AC_|/2 (i.e., χ must be finite). Furthermore, we can expect a change in *A*(*f*) to occur for f* ≈ 1/τ̅_av_.

**Figure 4:**
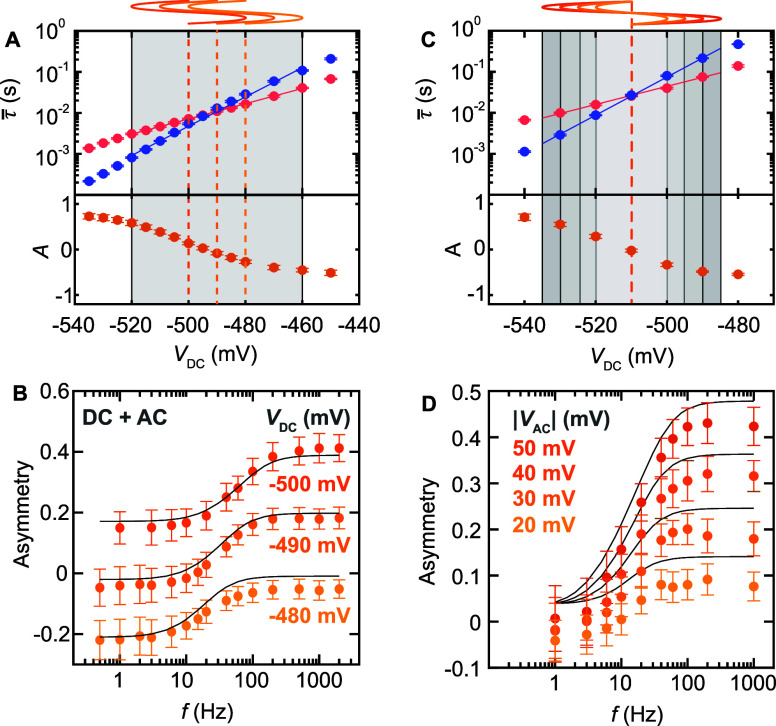
Frequency response in dependence of DC offset and AC amplitude. (A) Average residence times τ̅_low/high_ for Fe_low_ (red) and Fe_high_ (blue) and extracted asymmetry (lower panel) as a function of bias voltage *V*_DC_, with |*V*_AC_| = 0 mV. (B) Average state asymmetry *A* as a function of frequency with a constant |*V*_AC_| = 40 mV and three different V_DC_ values, as indicated in (A) with the yellow lines. (C) Average residence times τ̅_low/high_ for Fe_low_ (red) and Fe_high_ (blue) and extracted asymmetry (lower panel) as a function of bias voltage *V*_DC_, measured with *V*_AC_ = 0 mV. (D) Average state asymmetry *A* as a function of frequency, measured with a constant |*V*_DC_| = −510 mV and four different |*V*_AC_| values, as indicated in (C) with the gray shaded areas and the yellow lines. Solid black lines in (B) and (D) represent the simulated frequency response based on the rate-equation model and the exponential fits (solid lines in (A) and (C)). The tip height was stabilized on the substrate at *V*_DC_ = −400 mV and *I*_t_ = 100 pA.

To generalize the relationship between τ̅_low/high_(*V*_DC_|*V*_AC_ = 0) and the resulting frequency response, we studied the stochastic response of individual Fe atoms for different input signals. First, we apply an input voltage with a fixed |*V*_AC_| = 40 mV around three different values of *V*_DC_, as illustrated in Figure 4A. Each of the cases shows a similarly sized change of *A*(*f*) with the previously observed sigmoidal shape (Figure 4B). This is expected given the nearly constant value of χ_Fe_ in the voltage range of interest. However, the sign change of *A*(*V*_DC_) in Figure 4A introduces a vertical offset for the three cases. Additionally, a shift of the *A*(*f*) curves in Figure 4B along the frequency axis with varying *V*_DC_ values can be seen. This reflects the lower τ̅_low,high_ value of the system at higher *V*_DC_.

To further illustrate the tunability of the frequency response in the state occupation, we measured *A*(*f*) for various values of |*V*_AC_|. We probed the frequency response for |*V*_AC_| = 20, 30, 40, and 50 mV, while oscillating around the same bias point of *V*_DC_ = −510 mV, as indicated with the yellow lines above Figure 4C. We find that the total change of *A*(*f*) keeps increasing as |*V*_AC_| increases (Figure 4D). Interestingly, *A*(*f* ≪ f*) saturates around 0 for each |*V*_AC_|. These observations suggest that the system adopts an average *A* with an effective bias of *V*_eff_ ≈ *V*_DC_. The saturated *A*(*f* ≫ f*) indicates that the application of *V*_AC_ mimics the application of *V*_DC_, where *V*_eff_ shifts toward shorter τ̅_low/high_ with increasing *f*. The relation between the τ̅_low/high_(*V*_DC_) and the time-averaged frequency response was statistically confirmed by measuring the change of *A*(*f*) for multiple Fe atoms using similar conditions (see Figure S7). Additionally, we could obtain varying trends of *A*(*f*) by modifying the rate evolution with the tip position, *V*_DC_, and the atom type (see Figure S8). Each of these data sets could be reproduced by simulating *A*(*f*) based on a rate-equation model, as exemplified in Figure 4B,D.

## Conclusions

In conclusion, we studied the stochastic response of the orbital memory states derived from individual Fe and Co atoms to a sinusoidal drive. For individual Fe and Co atoms, we observed phase-synchronization for residence times that coincide with the inverse of the applied drive frequencies. This is manifested by a deviation from a Poisson distribution compared to the DC limit. Notably, this effect can be observed for frequencies much higher than the inverse average residence time. Based on stochastic modeling, we can relate the amplitude of the synchronization effect to the evolution of the switching rates as a function of bias voltage. As a second effect, we observed a change in the state favorability, namely, the asymmetry, as a function of frequency. In contrast to the response of individual Fe atoms, this effect is absent for individual Co atoms. Based on modeling and measuring the state favorability and lifetimes as a function of applied DC voltage, in the absence of a sinusoidal drive, we found that both the synchronization effect and the frequency-dependent asymmetry are derived from the underlying voltage-dependent transition rates. This is an important consideration when using a sinusoidal drive and the resultant change in asymmetry as a measure of the mean residence time of a two-state system, when the telegraph processes cannot be resolved. In other words, a time-averaged frequency response can be observed only if the modulated switching rates exhibit a significant asymmetry change in the modulation range of interest. If no change in asymmetry with frequency is observed, then the system may still show dynamics and synchronization in that frequency range. We showed that the asymmetry and residence times of single-atom orbital memory can be controlled by tuning the tip position, *V*_DC_, and atom type. This in turn enables tuning of the frequency response of orbital memory to an AC stimulus. The characteristic frequency is dictated by the transition rates and can be tuned by the injection current *I*_t_.^12^ The magnitude of the frequency response depends on χ in the modulation range and can be tuned by the choice of the atom type, tip position, *V*_DC_, and *V*_AC_. This is advantageous when probing multiwell behavior, as for example seen in the atomic Boltzmann machine. As coupled orbital memory exhibits multiwell behavior,^15^ these studies provide a starting basis for future studies where the stochastic response of multistate systems to complex waveforms can be interrogated. We expect coupling atoms that switch stochastically at different time scales to respond in multiple frequency regimes.

## Methods

We performed scanning tunneling microscopy and spectroscopy (STM/STS) using a home-built UHV-STM system^26^ (*p* < 5 × 10^–10^ mbar), operating at a temperature of *T* ≈ 7 K. All measurements were performed with electrochemically etched W tips. Prior to approaching black phosphorus (BP), these tips were dipped and characterized on a clean Au(111) surface. We prepared individual Fe and Co atoms adsorbed on BP by *in situ* cleaving a bulk BP crystal (HQ graphene) and (co)depositing Fe and Co while cooling the substrate to ∼30 K.

To combine *V*_DC_ and *V*_AC_ signals, we used the setup illustrated in Figure S2. The AC modulation was provided by an arbitrary waveform generator (Keysight 33600 A Series) and added to *V*_DC_ using an active adder. We note that the reported voltage values are overestimated by 0.48% due to a small attenuation factor from the adder. The combined signal and the current signal are each recorded using a multifunction input/output device (NI USB-6251) for data acquisition.

Switching events in the measured current trace were detected by defining a threshold current between two separated current levels (Figure S3A) after subtraction of a linear background and after further subtraction of an oscillating current contribution in the form of a sine wave for *V*_AC_ ≠ 0. The residence times between switching events τ_*i*,low/high_ and their statistical distribution were further used in the analysis. Figure S3B exemplarily shows histograms of τ_low/high_ for *V*_DC_ = −500 mV, *V*_AC_ = 0 mV. Their distribution is described by an exponential function with decay constant , where τ̅_low/high_ is the average residence time . This exponential distribution is characteristic of a Poisson point process.

To quantify uncertainties in the measurement of τ̅, we define a standard deviation σ of τ̅ by averaging random sets of 500 τ_*i*,low/high_ acquired under equivalent experimental conditions and calculating the standard deviation of these averaged values. Error bars throughout the paper represent 1σ.

## Data Availability

All data needed to evaluate the conclusions in the paper are present in the paper or the Supporting Information. Data for all figures presented in this study are available at Radboud Data Repository. The following software was used: Nanonis 4.5 and LabVIEW 2015 (experiments), Gwyddion 2.60 and Matlab R2019b (data processing), Jupyter 7.0 (data simulations), and Adobe Illustrator 27.8.1 (figure preparation).

## Supporting Information Available

Additional experimental details and methods, including supplementary text describing the rate-equation simulations and stochastic real-time data simulations, additional data statistics, experimental setup, telegraph noise analysis, detailed phase-resolved analysis, and additional figures (PDF)

## Abbreviations

AC: alternating current
BP: black phosphorus
DC: direct current
SR: stochastic resonance
STM: scanning tunneling microscopy
STS: scanning tunneling spectroscopy
UHV: ultrahigh vacuum
