# Supplementary material for: Stochastic Syncing in Sinusoidally Driven Atomic Orbital Memory
Source: ACS Nano. 2024 Jan 30;18(6):4840–6. doi: 10.1021/acsnano.3c09635 (PMC10867893; doi:10.1021/acsnano.3c09635)
Supplement: Supplementary file 1 — nn3c09635_si_001.pdf [file nn3c09635_si_001.pdf]

Supplementary Materials for

Stochastic syncing in sinusoidally driven atomic orbital memory

Werner M. J. van Weerdenburg[1,†], Hermann Osterhage[1,†], Ruben Christianen[1], Kira Junghans[1], Eduardo Domínguez[2], Hilbert J. Kappen[2], Alexander Ako Khajetoorians[1,*]

[1]*Institute for Molecules and Materials, Radboud University, 6525 AJ Nijmegen, the Netherlands*

[2] *Donders Institute for Neuroscience, Radboud University, 6525 AJ Nijmegen, the Netherlands*

*corresponding author: a.khajetoorians@science.ru.nl

†both authors contributed equally

S1. Simulations of state occupation for AC bias input

The transition rates in the orbital memory are strongly bias dependent [1, 2]. Accordingly, V_{AC} modulates the transition rates in the bistable orbital memory periodically. In order to simulate the state occupation of the orbital memory in the AC regime, we developed a model of a Poisson point process with transition rates changing periodically in time. The stepwise approach of simulating the system dynamics and time-averaged state occupations from this stochastic model will be described in the following.

First, we derive the voltage dependence of the transition rates $w_{i\to j}$ between the binary orbital memory states. Fig. S4A exemplarily shows the mean state lifetimes of a single Fe atom for varying DC bias V_{DC}. The transition rates are derived from the mean value of the measured state lifetimes:

$$w_{i\to j} = \frac{1}{\bar{\tau}_i},\ (i = \text{high/low}). \qquad \text{(S1)}$$

We perform a weighed exponential fit in order to derive a functional dependence of the transition rates, on the applied bias voltage (see Figure S4A):

$$w_{i\to j}(V) = a \cdot \exp(b|V|). \qquad \text{(S2)}$$

In the fitting procedure, the values of $w_{i\to j}$ for each bias value are weighed with the inverse of their squared standard deviation $\frac{1}{\sigma^2}$. The choice of an exponential function is purely phenomenological. We constrain the fit on the bias interval [V_{DC} - V_{AC}/2, V_{DC} + V_{AC}/2] that is relevant for simulating the AC response. Following the exponential bias dependence, we model the time dependent transition rates like

$$w_{i\to j}(t) = a\ \cdot \exp\left(b\left[V_{\mathrm{DC}} + \frac{1}{2}V_{\mathrm{AC}}\sin(2\pi f t)\right]\right). \qquad \text{(S3)}$$

The time evolution of the state occupation $n_i(t)$ is then calculated numerically, starting from an equal distribution of both states $\left(n_{\mathrm{low}}(0) = n_{\mathrm{high}}(0) = \frac{1}{2}\right)$:

$$n_i(t + \Delta t) = n_i(t) + \Delta t\frac{dn_i}{dt}, \qquad \text{(S4)}$$

with[3]

$$\frac{dn_i}{dt} = n_j(t)w_{j\to i}(t) - n_i(t)w_{i\to j}(t). \qquad \text{(S5)}$$

Fig. S4B shows the result of a simulation for an Fe atom on BP under experimental conditions as in Fig. 2A of the main paper with an applied AC frequency of f = 10 Hz. It can be seen that the

expectation value of n_i quickly converges to a steady state where it oscillates in time with frequency *f*. In order to deduce the time averaged state asymmetry

$$A = n_{\mathrm{low}} - n_{\mathrm{high}} \tag{S6}$$

shown in Fig. 2D, we calculate the average state occupations (broken lines) within two full periods of the AC signal in the equilibrium region (shaded area).

S2. Simulations of stochastic real-time data for AC bias input

The DC residence time statistics are accurately described by exponential distributions, corresponding to homogeneous Poisson point processes. The switching dynamics with fixed rates (constant voltage) can therefore be simulated as a repetition of two consecutive random steps, where exponentially distributed waiting times are generated with the state-dependent rates.

The non-homogeneous case, corresponding to a finite V_{AC}, is in principle not much more complex. A Poisson process with a time-dependent rate can be simulated by noticing that the integrated rate

$$I(t) = \int_0^t w_{i \to j}(\tau) d\tau \tag{S7}$$

is a random variable that is exponentially distributed according to $P(I) = \exp(-I)$. The idea is then to generate a random value for I and invert $I(t)$ to get a random sample of the residence time. As explained in the previous section, the switching rates $w_{i \to j}(t)$ are fitted to an exponential of the voltage signal (Eq. S3). For the AC case, the integrated rate function is inverted numerically via a lookup table. The fact that the rate is a periodic function of time is exploited for efficiency.

The resulting synthetic residence times are then analyzed in the same manner as the experimental data. Fig. S5 and S6 show that the synthetic stochastic data (black solid lines) reproduces the synchronization observed for both, the Fe and the Co atom (Fig.S5A, S6A), as well as the different frequency response in the phase-resolved state occupations (Fig. S5B, S6B). Furthermore, it can be seen that the simulation, which is solely based on DC switching rates, reproduces the experimentally measured phase-resolved switching rates very well, underlining the feasibility of the description as a Poisson process with varying transition rates.

S3. Data statistics

The tunability of the switching rates of the orbital memory states can provide a lot of variability to the frequency response of the system, as exemplified in Fig. 4. In Fig. S7, we present additional data sets on different Fe atoms, taken with identical measurement conditions as Fig. 2 and 3. We note that these measurements were not taken with an identical tip apex. In each case, small variations in the DC switching rates (A-C) impact the frequency dependent residence times (D-F), but the overall trend is consistent between all data sets.

Further variation was found by using different values of V_{DC} and I_t, as shown in the additional data sets in Fig. S8. Moreover, Fig. S8F demonstrates that an individual Co atom can exhibit a (small) frequency response when χ_{Co} is finite (compared to Fig. 2 and 3 in the manuscript).

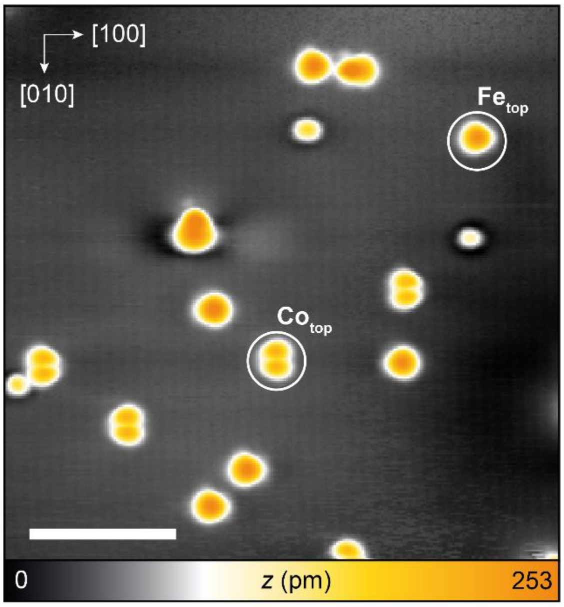

Figure S1: Constant-current STM image of the surface of black phosphorus with Co and Fe atoms adsorbed on the top binding site after deposition. To obtain well-isolated Fe and Co atoms, atoms can be laterally manipulated along the [010] direction and contaminated species[1] as well as BP vacancies[4] are avoided (V_{DC} = -400 mV, I_t = 10 pA, scale bar = 5 nm).

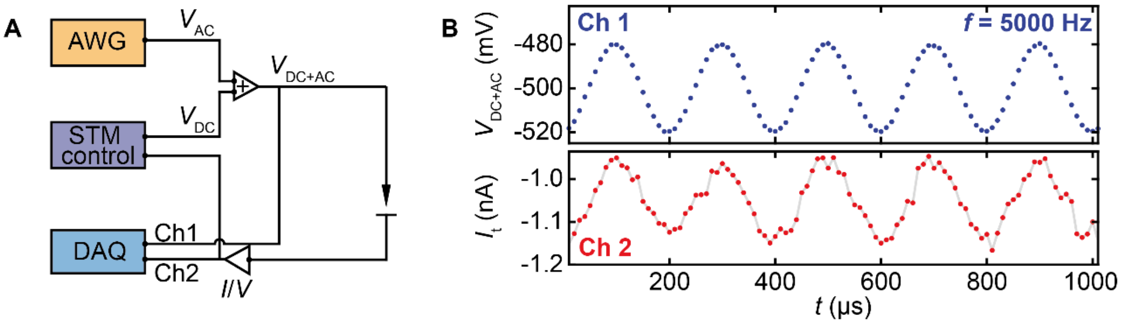

Figure S2: (A) Sketch of the experimental setup to measure with and without V_{AC}. (B) Example of a high-resolution time trace of V_{DC+AC} on Channel 1 with a frequency f = 5000 Hz and I_t on Channel 2, recorded with a sampling rate of 100000 samples/s for each channel. Note that the AC modulation has the correct amplitude ($|V_{AC}|$ = 40 mV) and that no significant phase delay is detected between the two channels at f = 5000 Hz.

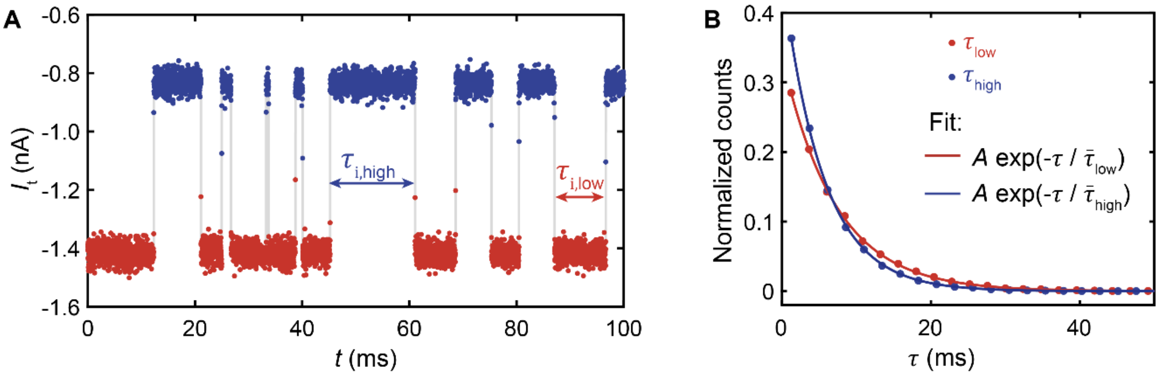

Figure S3: (A) Current trace of stochastic switching of a single Fe atom on BP measured with V_{DC} = -500 mV (V_{AC} = 0 mV). Single residence times $\tau_{i,\mathrm{low/high}}$ for the low and high state are exemplarily indicated. The tip height was stabilized on the substrate at V_{DC} = -400 mV and I_t = 100 pA. (B) Residence time histogram for Fe_{low} (red) and Fe_{high} (blue). The plots present a total of 28589 residence times, separated into 25 bins per state. Solid lines represent exponential functions with $\bar{\tau}$, where amplitude parameter A is fitted.

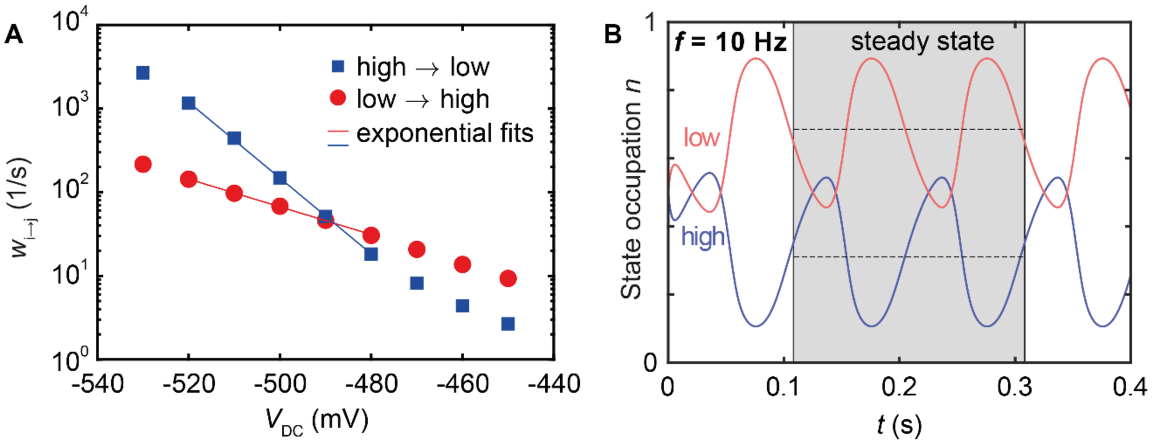

Figure S4: (A) DC transition rates $w_{i\to j}$ with exponential fits and (B) simulated state occupation as a function of time for an Fe atom on BP, according to the experimental conditions in Fig. 2C: V_{DC} = -500 mV, $|V_{AC}|$ = 40 mV (peak-to-peak), tip stabilized on the substrate at V_{DC} = -400 mV and I_t = 100 pA. Here, the state occupation in the case of f = 10 Hz is exemplarily shown. The simulation was initialized at t = 0 in $n_{\mathrm{low}} = n_{\mathrm{high}} = \frac{1}{2}$. The time-averaged occupations (broken lines) in equilibrium conditions are averaged over exactly two periods of the AC signal (shaded area).

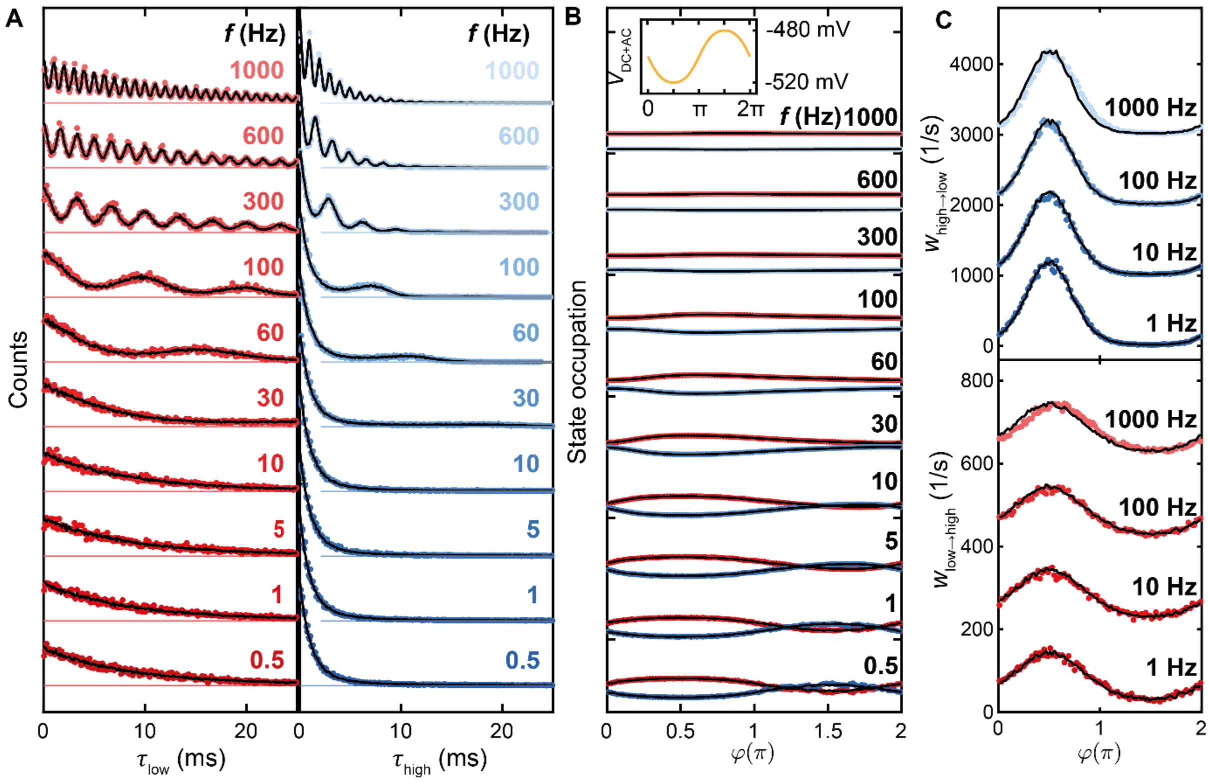

Figure S5: (A) Histograms of the residence times of Fe_{low} (red) and Fe_{high} (blue) for different frequencies (artificially offset). (B) Phase-resolved state occupation and (C) switching rate for different frequencies (artificially offset), averaged over many periods. The inset in (B) shows one period of the oscillatory bias voltage and defines the phase φ on the *x*-axis. Time-averaged properties of this data set are presented in Fig. 2D. The tip height was stabilized on the substrate at V_{DC} = -400 mV and I_t = 100 pA. Solid lines represent the results of stochastic simulations based on the DC switching rates $w_{i\to j}(V)$.

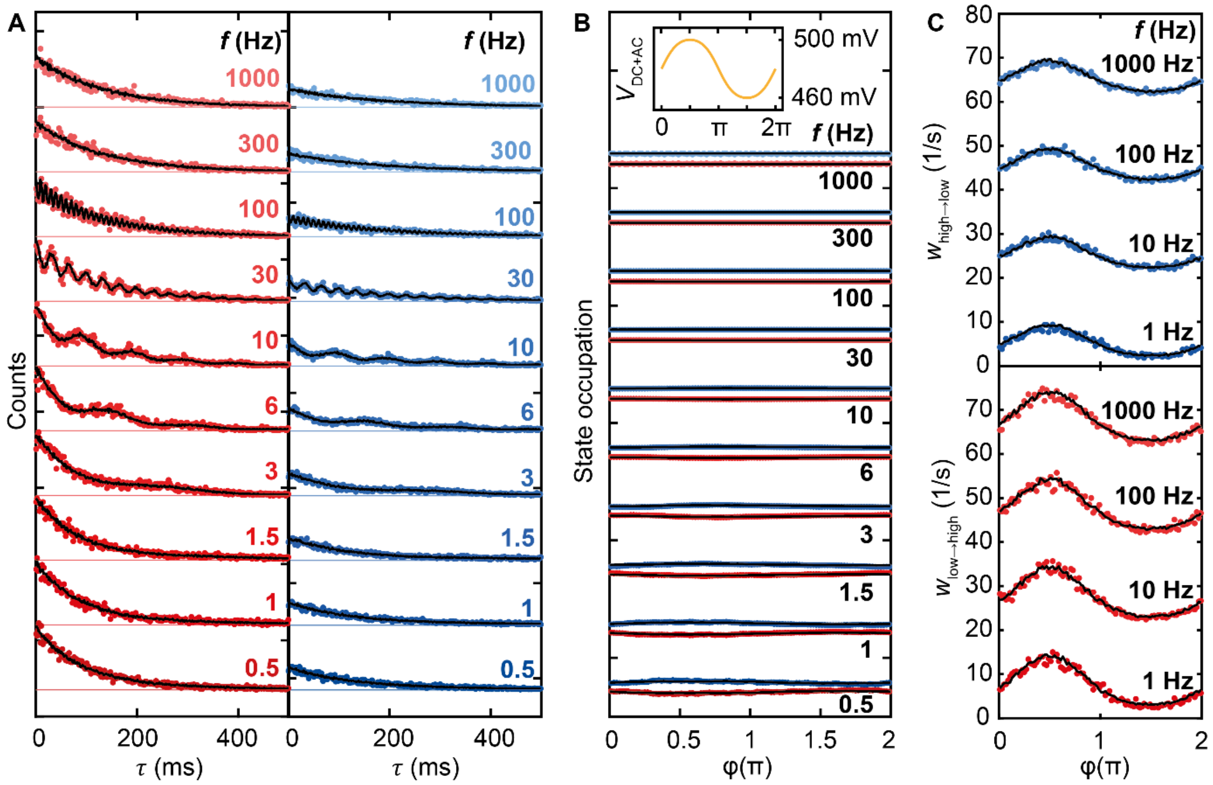

Figure S6: (A) Histograms of the residence times of Co_{low} (red) and Co_{high} (blue) for different frequencies (artificially offset). (B) Phase-resolved state occupation and (C) switching rate for different frequencies (artificially offset), averaged over many periods. The inset in (B) shows one period of the oscillatory bias voltage and defines the phase φ on the *x*-axis. Time-averaged properties of this data set are presented in Fig. 2D. The tip height was stabilized on the substrate at V_{DC} = -400 mV and I_t = 30 pA. Solid lines represent the results of stochastic simulations based on the DC switching rates $w_{i\to j}(V)$.

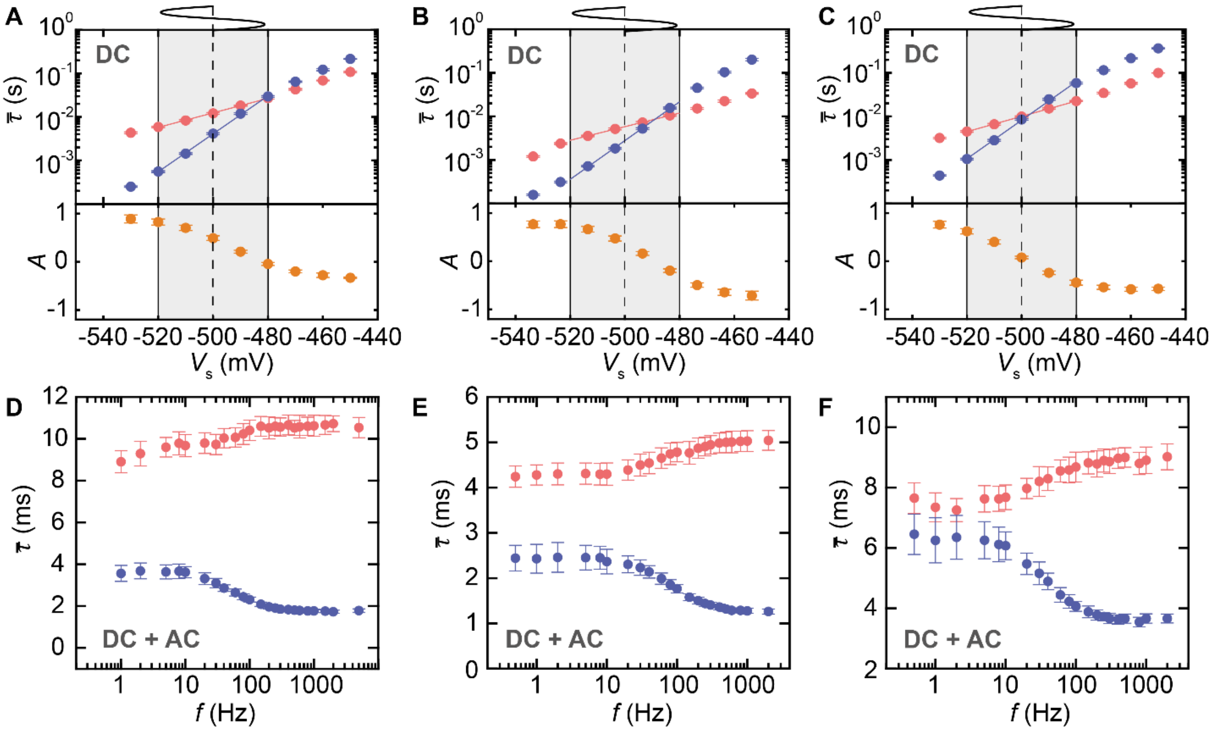

Figure S7: (A-C) Average residence times and extracted asymmetry, measured with V_{AC} = 0 mV for different Fe atoms and tips, and (D-F) the corresponding frequency response of the average residence times. The data sets are taken with measurement conditions comparable to the data set presented in Fig. 2A,C,D and Fig. 3A-C; each data set was measured with $|V_{AC}|$ = 40 mV and V_{DC} = -500 mV, and the tip height was stabilized on the substrate at V_{DC} = -400 mV and I_t = 100 pA.

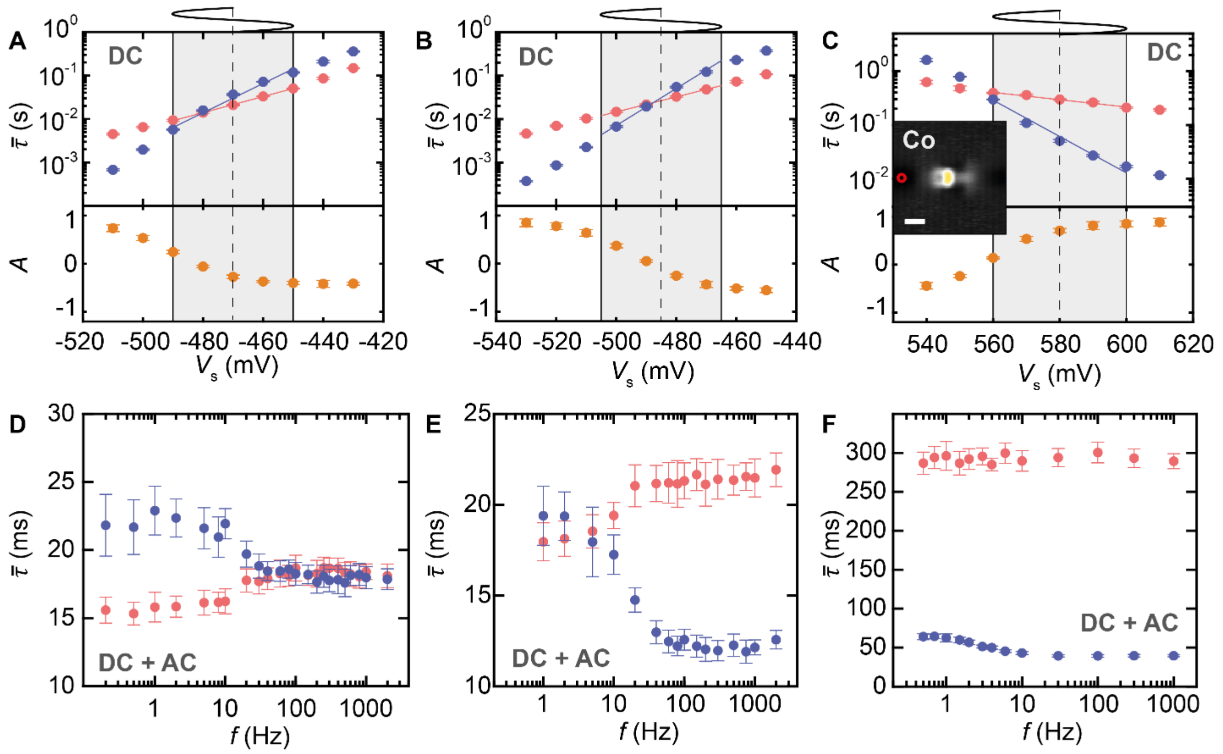

Figure S8: (A-C) Average residence times and extracted asymmetry (V_{AC} = 0 mV) and (D-F) the corresponding frequency response of the average residence times. These data sets were measured (A,D) on top of an individual Fe atom using V_{DC} = -470 mV, (B,E) on top of an individual Fe atom using V_{DC} = -485 mV, and (C,F) next to an individual Co atom using V_{DC} = 580 mV. Each data set was measured with $|V_{AC}|$ = 40 mV and with a different tip and atom. The tip height was stabilized on the substrate at V_{DC} = -400 mV and (A,D) I_t = 130 pA, (B,E) I_t = 100 pA (C,F) I_t = 70 pA.

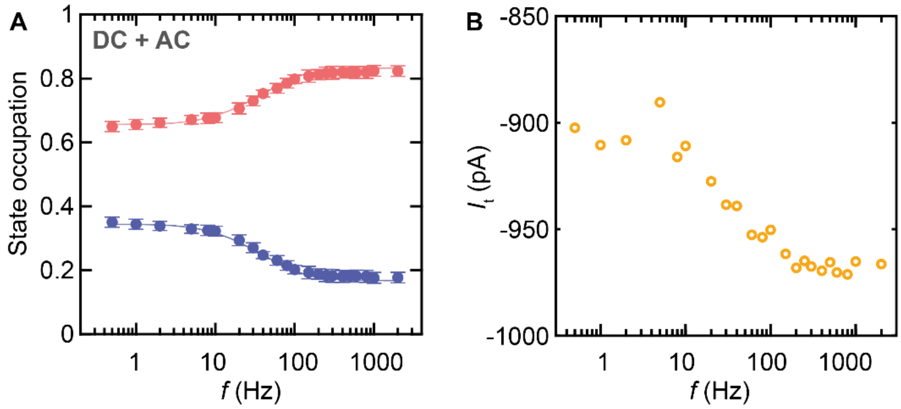

Figure S9: Comparison between the frequency-induced change in (A) the state occupation and (B) the time-averaged current. Data set corresponds to the data presented in Fig. 2(A,C,D) and Fig. 3(A-C).

References

(1) Kiraly, B.; Rudenko, A. N.; van Weerdenburg, W. M. J.; Wegner, D.; Katsnelson, M. I.; Khajetoorians, A. A. An orbitally derived single-atom magnetic memory. *Nat. Commun.* **2018**, *9*, 3904. DOI: 10.1038/s41467-018-06337-4.

(2) Kiraly, B.; Knol, E. J.; Rudenko, A. N.; Katsnelson, M. I.; Khajetoorians, A. A. Orbital memory from individual Fe atoms on black phosphorus. *Phys. Rev. Res.* **2022**, *4*, 033047. DOI: 10.1103/PhysRevResearch.4.033047.

(3) Löfstedt, R.; Coppersmith, S. N. Stochastic resonance: Nonperturbative calculation of power spectra and residence-time distributions. *Phys. Rev. E* **1994**, *49*, 4821-4831. DOI: 10.1103/PhysRevE.49.4821.

(4) Kiraly, B.; Hauptmann, N.; Rudenko, A. N.; Katsnelson, M. I.; Khajetoorians, A. A. Probing Single Vacancies in Black Phosphorus at the Atomic Level. *Nano Lett.* **2017**, *17*, 3607-3612. DOI: 10.1021/acs.nanolett.7b00766.
